# Supplementary material for: Endogenously Produced SARS-CoV-2 Specific IgG Antibodies May Have a Limited Impact on Clearing Nasal Shedding of Virus during Primary Infection in Humans
Source: Viruses. 2021 Mar 20;13(3):516. doi: 10.3390/v13030516 (PMC8003723; doi:10.3390/v13030516)
Supplement: Supplementary file 1 [file viruses-13-00516-s001.pdf]

Endogenously produced SARS-CoV-2 specific IgG antibodies may have a limited impact on clearing nasal shedding of virus during primary infection in humans

Shuyi Yang [1,2], Keith R Jerome [2,3], Alexander L. Greninger [2,3], Joshua T. Schiffer [2,4,5,*,†] and Ashish Goyal [2,*,†]

1 Department of Data Science, University of California San Diego, La Jolla, CA 92093, USA; s7yang@ucsd.edu
2 Vaccine and Infectious Disease Division, Fred Hutchinson Cancer Research Center, Seattle, WA 98109, USA
3 Department of Laboratory Medicine and Pathology, University of Washington School of Medicine, Seattle, WA 98195, USA; kjerome@fredhutch.org (K.R.J.); agrening@uw.edu (A.G.)
4 Clinical Research Division, Fred Hutchinson Cancer Research Center, Seattle, WA 98109, USA
5 Department of Medicine, University of Washington, Seattle, WA 98195, USA
* Correspondence: jschiffe@fredhutch.org (J.T.S.); agoyal@fredhutch.org (A.G.)
† These authors contributed equally.

Supplementary Information

S1. Simplification of the full model involving the development of humoral immunity

To capture the observed antibodies expansion dynamics, we developed a model based on the development of humoral immunity involving B cells as shown in **Figure S2**. Here, we assume that immature B cells are generated in bone marrow and they enter peripheral circulation as transitional B cells (B_T) at a constant rate λ_T, that are naturally cleared at rate δ_T. In this model, the transitional B cells turn into naïve B cells at rate r_C that are then activated without and with the help of T cells into marginal zone (MZ) B cells (B_Z) and follicular (FO) B cells (B_F) with fractions f and $1-f$, respectively [1]. Upon activation, marginal zone B cells and follicular B cells are assumed to proliferate at rates $r_{\phi Z}$ and $r_{\phi F}$, respectively, and saturate at rates $k_{\phi Z}$ and $k_{\phi F}$, respectively, while their natural clearance is denoted by δ_Z and δ_F, respectively. The clonally expanded marginal zone B cells and follicular B cells further differentiates into plasma cells, correspondingly becoming major contributors of IgM (I_M; short-lived response) and IgG (I_G) antibodies, respectively [2, 3]. IgM and IgG antibodies are produced at rates α_M and α_G, respectively, and are naturally cleared at rate d_M and d_G [4]. For the sake of simplification and since we are only modelling primary exposure, we ignore the differentiation of follicular B cells into memory B cells. The model is thus represented by the following system of ordinary differential equations,

$$\frac{dB_T}{dt} = \lambda_T - \delta_T B_T - r_C B_T$$
$$\frac{dB_Z}{dt} = f.r_C B_T + \frac{r_{\phi Z} B_Z}{k_{\phi Z} + B_Z} - \delta_Z B_Z$$
$$\frac{dB_F}{dt} = (1-f).r_C B_T + \frac{r_{\phi F} B_F}{k_{\phi F} + B_F} - \delta_F B_F$$
$$\frac{dI_M}{dt} = \alpha_M B_Z - d_M I_M$$
$$\frac{dI_G}{dt} = \alpha_G B_F - d_G I_G \quad (1)$$

Because of the time it takes to overcome negative selection pressure in the bone marrow to produce immature B cells, and time difference between viral recognition and antibody production in periphery, we assumed that IgM and IgG antibodies are not produced for the first τ_M and τ_G days after infection. In the absence of rich data, we reduce the dimensionality of the system by first assuming that transitional B cells are in quasi-steady state giving rise to $\overline{B_T} = \frac{\lambda_T}{\delta_T + r_C}$. Thus, the system of ordinary differential equations gets reduced to,

$$\frac{dB_Z}{dt} = f.r_c\overline{B_T} + \frac{r_{\phi Z}B_Z}{k_{\phi Z}+B_Z} - \delta_Z B_Z$$
$$\frac{dB_F}{dt} = (1-f).r_c\overline{B_T} + \frac{r_{\phi F}B_F}{k_{\phi F}+B_F} - \delta_F B_F$$
$$\frac{dI_M}{dt} = \alpha_M B_Z - d_M I_M$$
$$\frac{dI_G}{dt} = \alpha_G B_F - d_G I_G \qquad (2)$$

The parameter f is important to initially introduce the MZ and FO B cells, but in the absence of adequate data, we assume that it is the proliferation of these B cells that ramps up the response. Thus, to model the phase "post-activation" without the exact data about different B cell classes and to further simplify the system, we assumed $f.r_C\beta \ll \frac{r_{\phi Z}B_Z}{k_{\phi Z}+B_Z}$ and $(1-f).r_C\beta \ll \frac{r_{\phi F}B_F}{k_{\phi F}+B_F}$. Using the quasi-steady state, we have $B_Z = \frac{d_M I_M}{\alpha_M}$ and $B_F = \frac{d_G I_G}{\alpha_G}$. By substituting B_Z and B_F, our initial five-dimensional model (1) gets reduced to a simple 2-dimensional system (Model M3, Table S1) as,

$$\frac{dI_M}{dt} = \frac{r_M I_M}{k_M+I_M} - d_M I_M$$

$$\frac{dI_G}{dt} = \frac{r_G I_G}{k_G+I_G} - d_G I_G \qquad (3)$$

In this model, r_M, k_M, r_G, and k_G represent the production rate of IgM, the level of IgM antibodies at which its production rate becomes 50%, the production rate of IgG, the level of IgG antibodies at which its production rate becomes 50%, respectively. The transformative parameters are: $r_M = \frac{r_{\phi Z}\alpha_M}{d_M}$, $k_M = \frac{k_{\phi Z}\alpha_M}{d_M}$, $d_M = \delta_Z$, $r_G = \frac{r_{\phi F}\alpha_G}{d_G}$, $k_G = \frac{k_{\phi F}\alpha_G}{d_G}$ and $d_G = \delta_F$.

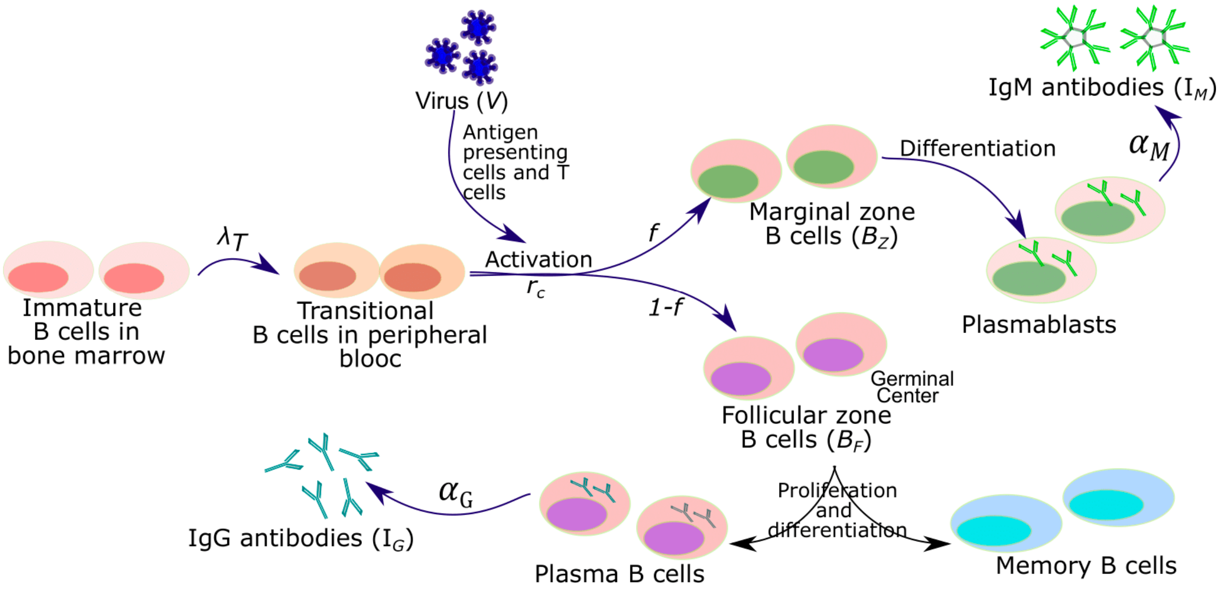

Figure S1: The schematic representation of the development of humoral immunity. In this model, immature B cells are generated in bone marrow and enter peripheral circulation as transitional B cells (B_T) at rate λ_T, that are naturally cleared at rate δ_T. The transitional B cells develop into naïve B cells at rate r_C that are then activated without and with the help of T cells into marginal zone (MZ) B cells (B_Z) and follicular (FO) B cells (B_F) with fractions f and $1-f$, respectively [1]. Upon activation, marginal

zone B cells and follicular B cells proliferate at rates $r_{\phi Z}$ and $r_{\phi F}$, respectively, and saturate at rates $k_{\phi Z}$ and $k_{\phi F}$, while their natural clearance is denoted by δ_Z and δ_F. The clonally expanded marginal zone B cells and follicular B cells further differentiates into plasma cells, correspondingly becoming major contributors of IgM (I_M; short-lived response) and IgG (I_G) antibodies, respectively [2, 3]. IgM and IgG antibodies are produced at rates α_M and α_G, respectively, and are naturally cleared at rate d_M and d_G [4].

Table S1: List of models fitted to longitudinal data of IgG and IgM antibodies from 6 severe and 20 non-severe patients. Here, AIC represents Akaike Information criteria and -2LL represents -2log-likelihood. Models with smaller AIC are better supported by the experimental data. In the model, we have equations for marginal zone (MZ) B cells (B_Z) and follicular (FO) B cells (B_F). Upon activation, marginal zone B cells and follicular B cells proliferate at rates $r_{\phi Z}$ and $r_{\phi F}$, respectively, and saturate at rates $k_{\phi Z}$ and $k_{\phi F}$, while their natural clearance is denoted by δ_Z and δ_F. The clonally expanded marginal zone B cells and follicular B cells further differentiate into plasma cells, correspondingly becoming major contributors of IgM (I_M; short-lived response) and IgG (I_G) antibodies, respectively. Moreover, IgM and IgG antibodies are produced at rates α_M and α_G, respectively, and are naturally cleared at rate d_M and d_G. The best model is shown in red. The analytical solution for $I_G(t)$ under models M1, M2 and M3 are $I(t) = I(0)e^{-d_M t} + \frac{R_m}{d_M}(1 - e^{-d_M t})$, $I_M(t) = I_M(0)e^{(r_M - d_M)t}$ and $|R_G - d_G k_G - d_G I_G(t)|^{-\frac{A}{d_G}} \; |I_G(t)|^B = e^t \; |R_G - d_G k_G - d_G I_G(0)|^{-\frac{A}{d_G}} \; |I_G(0)|^B$, where $B = \frac{k_G}{(R_G - d_G k_G)}$ and $A = 1 + \frac{d_G k_G}{(R_G - d_G k_G)}$. The analytical solution for $I_M(t)$ under models M1, M2 and M3 is similar and can be obtained by substituting "*G*" subscripts by "*M*".

Model	Model formation	Additional constraints on parameters	AIC (-2LL)
M1	$\frac{dB_Z}{dt} = r_{\phi Z} - \delta_Z B_Z$<br>$\frac{dI_M}{dt} = \alpha_M B_Z - d_M I_M$<br>$\frac{dB_F}{dt} = r_{\phi F} - \delta_F B_F$<br>$\frac{dI_G}{dt} = \alpha_G B_F - d_G I_G$<br>Using quasi-steady state, we convert this 4-dimensional system into the following two-dimensional system<br>$\frac{dI_M}{dt} = r_M - d_M I_M$<br>$\frac{dI_G}{dt} = r_G - d_G I_G$<br>Additionally, we have<br>$r_i = d_i I_i(0)$ for $t \le \tau_i$, $i \in [M, G]$<br>When $t > \tau_i, r_i = R_i, i \in [M, G]$	• 8 unknown parameters: $r_M, I_{M0}, d_M, \tau_M, r_G, I_{G0}, d_G, \tau_G$<br>• Estimated random effects of all parameters besides d_M and d_G<br>• Error model: constant (IgG), combined2 (IgM)<br>• $corr(\tau M, \tau G)$ = 0.60	807.3<br>771.3
M2	$\frac{dB_Z}{dt} = r_{\phi Z} B_Z - \delta_Z B_Z$	• 8 unknown parameters: $r_M, I_{M0}, d_M, \tau_M, r_G, I_{G0}, d_G, \tau_G$	1036.5<br>1004.5

	$\frac{dI_M}{dt} = \alpha_M B_Z - d_M I_M$<br>$\frac{dB_F}{dt} = r_{\phi F} B_F - \delta_F B_F$<br>$\frac{dI_G}{dt} = \alpha_G B_F - d_G I_G$<br>Using quasi-steady state, we convert this 4-dimensional system into the following two-dimensional system<br>$\frac{dI_M}{dt} = r_M I_M - d_M I_M$<br>$\frac{dI_G}{dt} = r_G I_M - d_G I_G$<br>Additionally, we have<br>$r_i = d_i$ for $t \leq \tau_i$, $i \in [M, G]$<br>When $t > \tau_i, r_i = R_i, i \in [M, G]$	• Estimated random effects of all parameters besides d_M and d_G<br>• Error model: constant (IgG), constant (IgM)<br>• No correlations among parameters.	
M3	$\frac{dB_Z}{dt} = \frac{r_{\phi Z} B_Z}{k_Z + B_Z} - \delta_Z B_Z$<br>$\frac{dI_M}{dt} = \alpha_M B_Z - d_M I_M$<br>$\frac{dB_F}{dt} = \frac{r_{\phi F} B_F}{k_Z + B_F} - \delta_F B_F$<br>$\frac{dI_G}{dt} = \alpha_G B_F - d_G I_G$<br>Using quasi-steady state, we convert this 4-dimensional system into two-dimensional system<br>$\frac{dI_M}{dt} = \frac{r_M I_M}{k_M + I_M} - d_M I_M$<br>$\frac{dI_G}{dt} = \frac{r_G I_G}{k_G + I_G} - d_G I_G$<br>Additionally, we have<br>$r_i = d_i[k_i + I_i(0)]$ for $t \leq \tau_i$, $i \in [M, G]$<br>When $t > \tau_i, r_i = R_i, i \in [M, G]$	• 10 unknown parameters: $r_M, \log_{10} I_{M0}, d_M, \log_{10} \tau_M, k_M, r_G, \log_{10} I_{G0}, d_G, \log_{10} \tau_G, k_G$<br>• Estimated random effects of all parameters besides d_M, d_G, k_M and k_G<br>• Error model: constant (IgG), proportional (IgM)<br>• $corr(r_M, r_G, \log_{10} \tau_G) = 0.16, -0.37, 0.43$ in the order of (r_M and r_G, r_G and $\log_{10} \tau_G$, r_M and $\log_{10} \tau_G$)<br>• $corr(\log_{10} I_{M0}, \log_{10} \tau_M) = 0.56$.	800.36<br>756.36

Table S2: Estimated parameters under the model that best recapitulated the longitudinal IgG and IgM data from 26 patients (i.e., model M3 in **Table S1**). Severity index of 0 and 1 denotes non-severe and severe cases, respectively. Parameters d_M, d_G, k_M and k_G are estimated at 0.48/day, 0.26/day, 7.4×10^{-5} SCO and 1.3 SCO, respectively.

ID	r_M (SCO/day)	$\log_{10} I_{M0}$ (SCO)	r_G (SCO/day)	$\log_{10} I_{G0}$ (SCO)	$\log_{10} \tau_G$ (days)	$\log_{10} \tau_M$ (days)	Severity
1	0.51	-0.65	71.69	-0.33	0.53	0.77	0
2	3.27	-0.55	24.71	-0.32	0.57	0.71	1
3	0.65	-0.25	37.34	-0.33	0.58	1.09	1
4	0.81	-0.68	2.90	-0.43	0.62	0.63	0
5	1.87	-0.59	6.82	-0.56	0.82	0.79	1
6	1.04	-0.32	10.65	-0.20	0.79	1.20	0
7	1.38	-0.61	2.88	-0.41	0.83	0.88	0
8	1.03	-0.77	1.13	-0.13	0.85	0.48	0

9	1.24	-0.71	5.07	-0.55	0.84	0.75	0
10	2.17	-0.46	13.50	-0.33	0.96	0.99	0
11	0.86	-0.27	1.16	-0.32	0.96	1.21	0
12	7.50	-0.30	3.09	-0.30	0.94	1.05	1
13	4.35	-0.43	8.49	-0.24	0.99	1.03	0
14	2.95	-0.61	5.59	-0.29	1.09	1.10	0
15	2.72	-0.49	3.26	-0.21	1.06	0.95	1
16	0.74	-0.28	3.46	-0.31	0.90	1.16	0
17	22.62	-0.36	9.25	-0.42	1.09	1.01	0
18	2.06	-0.31	3.87	-0.42	1.08	0.99	0
19	1.54	-0.28	1.49	-0.40	1.12	1.19	0
20	13.22	-0.50	7.03	0.07	1.30	0.92	1
21	1.12	-0.39	1.49	-0.35	1.13	1.11	0
22	1.98	-0.50	1.22	-0.36	1.17	0.93	0
24	3.77	-0.48	2.44	-0.61	1.05	1.04	0
25	0.25	-0.43	0.76	-0.22	0.93	1.12	0
26	0.86	-0.24	0.66	-0.45	1.01	1.06	0
27	3.27	-0.57	2.54	-0.32	1.05	0.81	0
**Mean**	3.22	-0.46	8.94	-0.34	0.93	0.96	NA
**Median**	1.71	-0.47	3.36	-0.33	0.96	1.00	NA
**Standard deviation**	4.78	0.16	15.18	0.14	0.20	0.19	NA
**95% CI**	[1.23, 2.97]	[-0.58, -0.42]	[2.47, 6.66]	[-0.42, -0.24]	[0.85, 1.0]	[0.85, 1.0]	

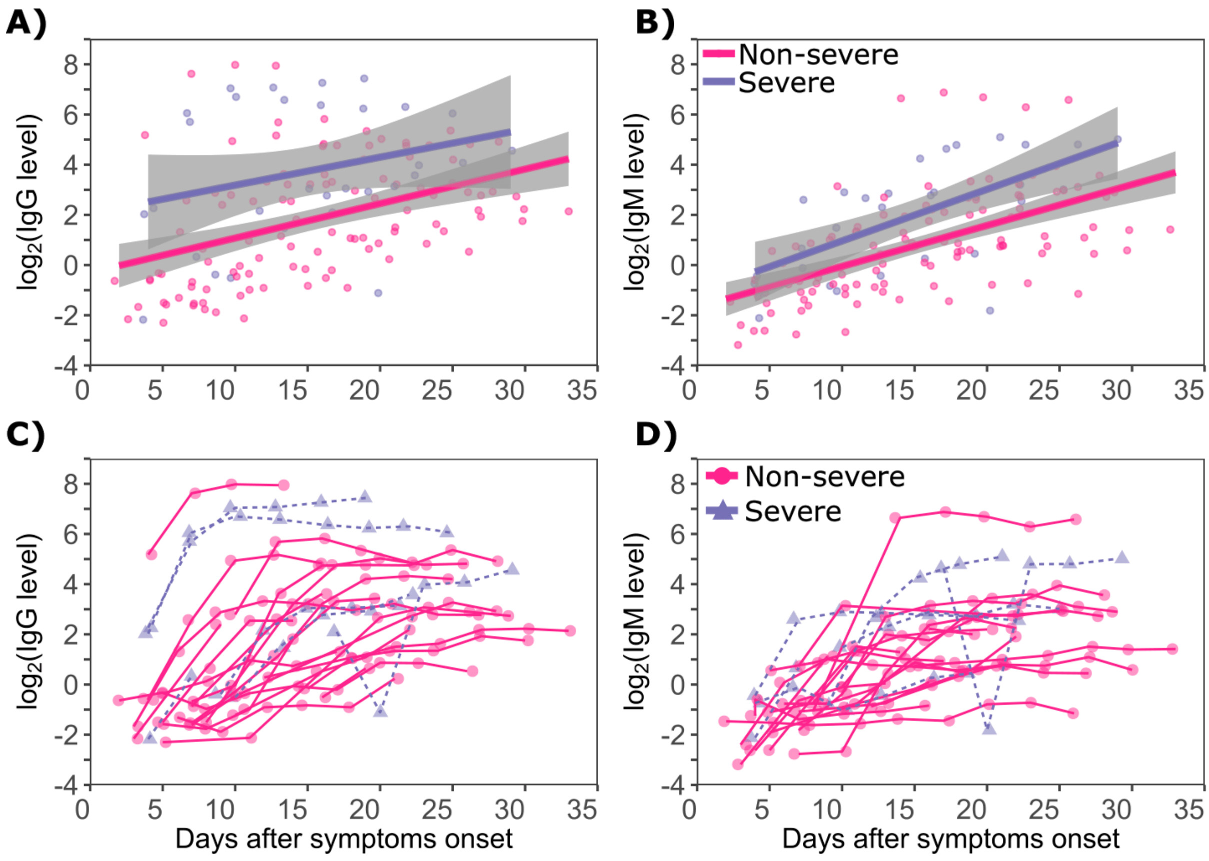

Figure S2: (A)-(B): The data grouped by severity index, severe patients (blue) and non-severe patients (pink). Linear regression was applied to describe the general trend of the IgG and IgM antibody level change over time for all 26 patients by severity. **(C)-(D):** Longitudinal patterns of IgG and IgM antibodies for all 26 patients.

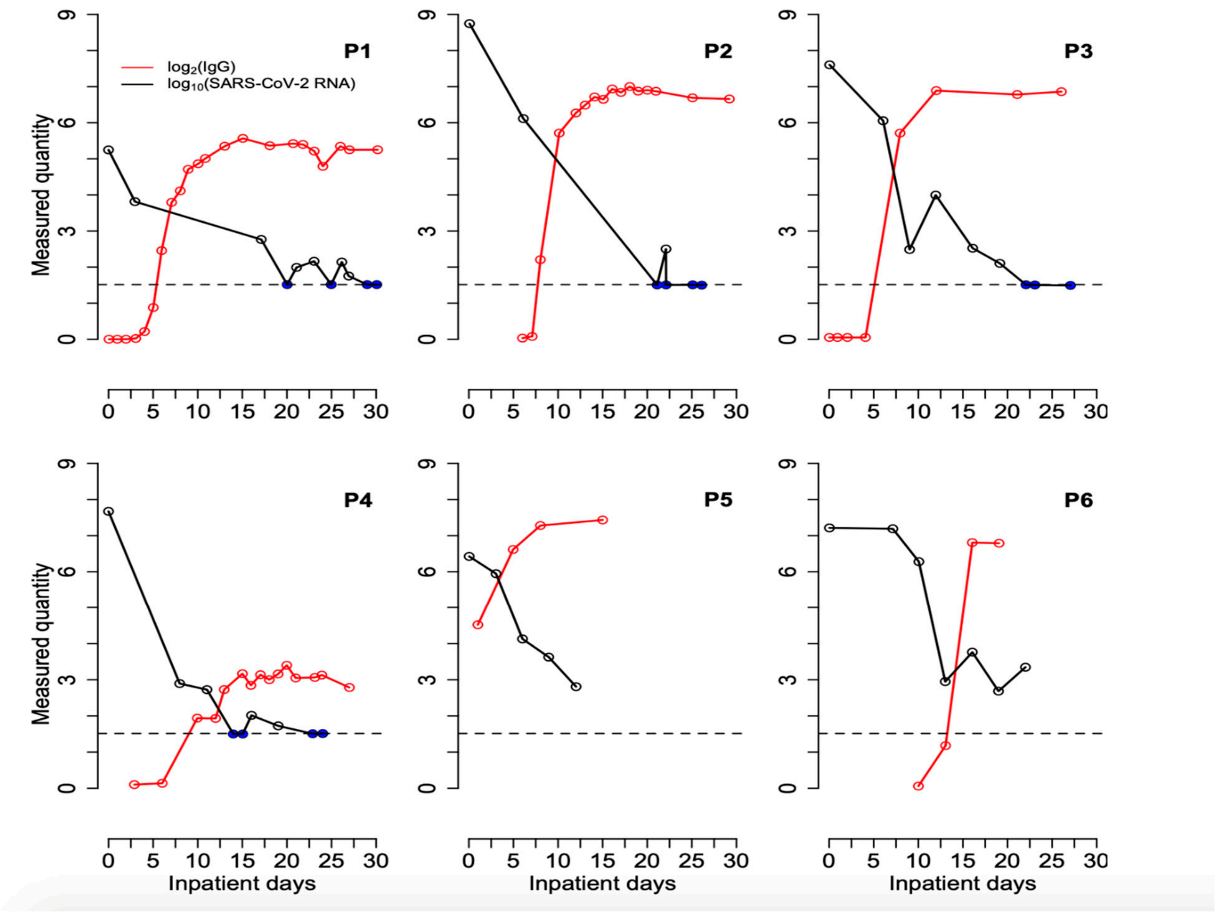

Figure S3: Observed longitudinal dynamics of SARS-CoV-2 viral loads (black) and IgG antibodies (red) data of each patient. Markers represent the observed data and the limit of detection is 32 copies/mL. Initially the viral loads were measured in cycle threshold and later converted into copies. The observation period starts on the first day of hospitalization (i.e., inpatient days), which is also the day of first positive RT-PCR.

Table S3: Group MP, MQ, MR, MS and MT represent the class of models that capture 5 different possible ways in which antibody can affect viral replication. MP, MQ, MR, MS and MT represent the case where antibodies do not affect viral replication, antibodies bind to the virus, antibodies inhibit viral entry, antibodies inhibit viral entry as well as bind to the virus and antibodies facilitate the death of infected cells. In each group, subgroups v1-v3 correspond to 3 antibody generation saturation mechanisms of one-off stimulation on B cells, v4-v6 correspond to 3 mechanisms of continuous stimulation on B cells, and v7-v9 correspond to 3 mechanisms of delayed continuous stimulation on B cells. The viral dynamics model includes SARS-CoV-2 (V) that infects susceptible cells (T) at rate β and converts them to infected cells (I). Infected cells are cleared by innate response in a density dependent manner at rate $\delta I I^k$. SARS-CoV-2 virions are produced by infected cells at rate p, and the virus is naturally cleared at rate c. To recapitulate IgG antibodies dynamics, we borrowed models from Table S1 and explored different ways in which viral load can impact antibody generation. The best performing model dictates that after the first τ days, IgG antibodies level (I_G) begins to grow at rate r_G, and it is saturated by a high level of IgG antibody (controlled by k_G). The natural clearance rate of IgG antibodies is denoted by d_G. The initial conditions are $T(t_{zero}) = 10^7$ cells/mL, $I(t_{zero}) = 1$ cells/mL and

$V(t_{zero}) = \frac{pI(t_{zero})}{c}$. The best model is shown in red. Some parameters were fixed: c = 15/day, k = 0.09, $\beta = 5.9 \times 10^{-8}$ virions^{-1}day^{-1} [5]. As the data on the upslope of viral dynamics is missing in these patients, we fixed the fixed effects of viral dynamics parameters such as $log10p = 2.59$/day, δ = 3.14/day, $log10\beta = -7.23$ virions^{-1}.day^{-1}, $log10\big(T(t_{zero})\big) = 7$ cells and $k = 0.09$ that were obtained from a previously published study in which few patients had upslope data [5].

**Model**	**Model formation**	**Additional constraints on parameters**	**AIC (-2LL)**
MP-v1	IgG doesn't affect VL and there is one-time stimulation of B cells,<br>$\frac{dT}{dt} = -\beta VT$<br>$\frac{dI}{dt} = \beta VT - \delta I I^k$<br>$\frac{dV}{dt} = pI - cV$<br>$\frac{dI_G}{dt} = r_G - d_G I_G$<br>Initial conditions:<br>$r_G = d_G I_G(0)$ if $t < \tau_G$, else $r_G = R_G$	• Estimated fixed and random effects of parameters: $\log_{10} I_{G0}, r_G, \log_{10}\tau_G, t_{zero}$<br>• Estimated only fixed effects of parameters: d_G<br>• Estimated only random effects of parameters $\log_{10} p, \delta, \log_{10}\beta, k, log10(T(t_{zero}))$<br>• Error model: constant (IgG), constant (VL)<br>• No correlations among parameters	231.4<br>199.4
MP-v2	IgG doesn't affect VL and there is one-time stimulation of B cells, which gets saturated at the high concentration of IgG,<br>$\frac{dT}{dt} = -\beta VT$<br>$\frac{dI}{dt} = \beta VT - \delta I I^k$<br>$\frac{dV}{dt} = pI - cV$<br>$\frac{dI_G}{dt} = \frac{r_G I_G}{k_G + I_G} - d_G I_G$<br>Initial conditions:<br>$r_G = d_G(k_G + I_G(0))$ if $t < \tau_G$, else $r_G = R_G$	• Estimated fixed and random effects of parameters: $\log_{10} I_{G0}, r_G, \log_{10}\tau_G, t_{zero}$<br>• Estimated only fixed effects of parameters: k_G, d_G<br>• Estimated only random effects of parameters $\log_{10} p, \delta, \log_{10}\beta, k, log10(T(t_{zero}))$<br>• Error model: combined1 (IgG), constant (VL)<br>• Corr(τ_G, δ)= -0.995	195.6<br>157.6
MP-v3	IgG doesn't affect VL and there is one-time stimulation of B cells, which gets saturated at the high concentration of viral loads,<br>$\frac{dT}{dt} = -\beta VT$<br>$\frac{dI}{dt} = \beta VT - \delta I I^k$<br>$\frac{dV}{dt} = pI - cV$<br>$\frac{dI_G}{dt} = \frac{r_G I_G}{k_G + V} - d_G I_G$<br>Initial conditions:	• Estimated fixed and random effects of parameters: $\log_{10} I_{G0}, r_G, \log_{10}\tau_G, t_{zero}$<br>• Estimated only fixed effects of parameters: k_G, d_G<br>• Estimated only random effects of parameters $\log_{10} p, \delta, \log_{10}\beta, k, log10(T(t_{zero}))$<br>• Error model: constant	446.0<br>412.0

	$r_G = d_G(k_G + V(0))$ if $t < \tau_G$, else $r_G = R_G$	(IgG), constant (VL)<br>• No correlations among parameters	
MP-v4	IgG doesn't affect VL and there is continuous stimulation of B cells,<br>$$\frac{dT}{dt} = -\beta VT$$<br>$$\frac{dI}{dt} = \beta VT - \delta I I^k$$<br>$$\frac{dV}{dt} = pI - cV$$<br>$$\frac{dI_G}{dt} = r_G V - d_G I_G$$<br>Initial conditions:<br>$r_G = \frac{d_G I_G(0)}{V(0)}$ if $t < \tau_G$, else $r_G = R_G$	• Estimated fixed and random effects of parameters: $\log_{10} I_{G0}, r_G, \log_{10} \tau_G, t_{zero}$<br>• Estimated only fixed effects of parameters: d_G<br>• Estimated only random effects of parameters $\log_{10} p, \delta, \log_{10} \beta, k, log10(T(t_{zero}))$<br>• Error model: constant (IgG), constant (VL)<br>• No correlations among parameters	387.7<br>355.7
MP-v5	IgG doesn't affect VL and there is continuous stimulation of B cells, which gets saturated at the high concentration of IgG,<br>$$\frac{dT}{dt} = -\beta VT$$<br>$$\frac{dI}{dt} = \beta VT - \delta I I^k$$<br>$$\frac{dV}{dt} = pI - cV$$<br>$$\frac{dI_G}{dt} = \frac{r_G I_G V}{k_G + I_G} - d_G I_G$$<br>Initial conditions:<br>$r_G = \frac{d_G(K_G + I_G(0))}{V(0)}$ if $t < \tau_G$, else $r_G = R_G$	• Estimated fixed and random effects of parameters: $\log_{10} I_{G0}, r_G, \log_{10} \tau_G, t_{zero}$<br>• Estimated only fixed effects of parameters: k_G, d_G<br>• Estimated only random effects of parameters $\log_{10} p, \delta, \log_{10} \beta, k, log10(T(t_{zero}))$<br>• Error model: constant (IgG), constant (VL)<br>• No correlations among parameters	366.6<br>332.6
MP-v6	IgG doesn't affect VL and there is continuous stimulation of B cells, which gets saturated at high viral loads,<br>$$\frac{dT}{dt} = -\beta VT$$<br>$$\frac{dI}{dt} = \beta VT - \delta I I^k$$<br>$$\frac{dV}{dt} = pI - cV$$<br>$$\frac{dI_G}{dt} = \frac{r_G I_G V}{k_G + V} - d_G I_G$$<br>Initial conditions:<br>$r_G = \frac{d_G(K_G + V(0))}{V(0)}$ if $t < \tau_G$, else $r_G = R_G$	• Estimated fixed and random effects of parameters: $\log_{10} I_{G0}, r_G, \log_{10} \tau_G, t_{zero}$<br>• Estimated only fixed effects of parameters: k_G, d_G<br>• Estimated only random effects of parameters $\log_{10} p, \delta, \log_{10} \beta, k, log10(T(t_{zero}))$<br>• Error model: constant (IgG), constant (VL)<br>• No correlations among parameters	370.4<br>336.4

		•	
MP-v7	IgG doesn't affect VL, IgG response to infection is τ days delayed and there is continuous stimulation of B cells, $$\frac{dT}{dt} = -\beta VT$$ $$\frac{dI}{dt} = \beta VT - \delta II^k$$ $$\frac{dV}{dt} = pI - cV$$ $$\frac{dI_G}{dt} = r_G V(t-\tau) - d_G I_G$$	• Estimated fixed and random effects of parameters: $\log_{10} I_{G0}, r_G, \log_{10}\tau, t_{zero}$<br>• Estimated only fixed effects of parameters: d_G<br>• Estimated only random effects of parameters $\log_{10} p, \delta, \log_{10}\beta, k, log10(T(t_{zero}))$<br>• Error model: constant (IgG), constant (VL)<br>• No correlations among parameters	291.3<br>259.3
MP-v8	IgG doesn't affect VL, IgG response to infection is τ days delayed and there continuous stimulation of B cells, which gets saturated at high levels of IgG, $$\frac{dT}{dt} = -\beta VT$$ $$\frac{dI}{dt} = \beta VT - \delta II^k$$ $$\frac{dV}{dt} = pI - cV$$ $$\frac{dI_G}{dt} = \frac{r_G I_G V(t-\tau)}{k_G + I_G} - d_G I_G$$	• Estimated fixed and random effects of parameters: $\log_{10} I_{G0}, r_G, \log_{10}\tau, t_{zero}$<br>• Estimated only fixed effects of parameters: k_G, d_G<br>• Estimated only random effects of parameters $\log_{10} p, \delta, \log_{10}\beta, k, log10(T(t_{zero}))$<br>• Error model: constant (IgG), constant (VL)<br>• No correlations among parameters	271.8<br>237.8
MP-v9	IgG doesn't affect VL, IgG response to infection is τ days delayed and there is continuous stimulation of B cells, which gets saturated at high levels of viral loads, $$\frac{dT}{dt} = -\beta VT$$ $$\frac{dI}{dt} = \beta VT - \delta II^k$$ $$\frac{dV}{dt} = pI - cV$$ $$\frac{dI_G}{dt} = \frac{r_G I_G V(t-\tau)}{k_G + V} - d_G I_G$$	• Estimated fixed and random effects of parameters: $\log_{10} I_{G0}, r_G, \log_{10}\tau, t_{zero}$<br>• Estimated only fixed effects of parameters: d_G<br>• Estimated only random effects of parameters $\log_{10} p, \delta, \log_{10}\beta, k, log10(T(t_{zero}))$<br>• Error model: constant (IgG), constant (VL)<br>• No correlations among parameters	448.8<br>414.8
MQ-v1	IgG binds to virus in density-dependent manner and there is one-time stimulation of B cells, $$\frac{dT}{dt} = -\beta VT$$	• Estimated fixed and random effects of parameters: $\log_{10} I_{G0}, r_G, \log_{10}\tau_G, t_{zero}, \nu$<br>• Estimated only fixed	234.5<br>198.5

	$$\frac{dI}{dt} = \beta T - \delta I I^k$$ $$\frac{dV}{dt} = pI - cV - \nu I_G V$$ $$\frac{dI_G}{dt} = r_G - d_G I_G$$ Initial conditions:<br>$r_G = d_G I_G(0)$ if $t < \tau_G$, else $r_G = R_G$	effects of parameters: d_G<br>• Estimated only random effects of parameters $\log_{10} p, \delta, \log_{10} \beta, k, log10(T(t_{zero}))$<br>• Error model: constant (IgG), constant (VL)<br>• No correlations among parameters	
MQ-v2	IgG binds to virus in density-dependent manner and there is one-time stimulation of B cells, which gets saturated at high levels of IgG, $$\frac{dT}{dt} = -\beta V T$$ $$\frac{dI}{dt} = \beta V T - \delta I I^k$$ $$\frac{dV}{dt} = pI - cV - \nu I_G V$$ $$\frac{dI_G}{dt} = \frac{r_G I_G}{k_G + I_G} - d_G I_G$$ Initial conditions:<br>$r_G = d_G(k_G + I_G(0))$ if $t < \tau_G$, else $r_G = R_G$	• Estimated fixed and random effects of parameters:<br>$\log_{10} I_{G0}, r_G, \log_{10} \tau_G, t_{zero}, \nu$<br>• Estimated only fixed effects of parameters: k_G, d_G<br>• Estimated only random effects of parameters $\log_{10} p, \delta, \log_{10} \beta, k, log10(T(t_{zero}))$<br>• Error model: combined1 (IgG), constant (VL)<br>• Corr(τ_G, δ) = -0.999	207.6<br>165.6
MQ-v3	IgG binds to virus in density-dependent manner and there is one-time stimulation of B cells, which gets saturated at high levels of viral loads, $$\frac{dT}{dt} = -\beta V T$$ $$\frac{dI}{dt} = \beta V T - \delta I I^k$$ $$\frac{dV}{dt} = pI - cV - \nu I_G V$$ $$\frac{dI_G}{dt} = \frac{r_G I_G}{k_G + V} - d_G I_G$$ Initial conditions:<br>$r_G = d_G(k_G + V(0))$ if $t < \tau_G$, else $r_G = R_G$	• Estimated fixed and random effects of parameters:<br>$\log_{10} I_{G0}, r_G, \log_{10} \tau_G, t_{zero}, \nu$<br>• Estimated only fixed effects of parameters: k_G, d_G<br>• Estimated only random effects of parameters $\log_{10} p, \delta, \log_{10} \beta, k, log10(T(t_{zero}))$<br>• Error model: constant (IgG), constant (VL)<br>No correlations among parameters	413.3<br>375.3
MQ-v4	IgG binds to virus in density-dependent manner and there is continuous stimulation of B cells, $$\frac{dT}{dt} = -\beta V T$$ $$\frac{dI}{dt} = \beta V T - \delta I I^k$$ $$\frac{dV}{dt} = pI - cV - \nu I_G V$$ $$\frac{dI_G}{dt} = r_G V - d_G I_G$$	• Estimated fixed and random effects of parameters:<br>$\log_{10} I_{G0}, r_G, \log_{10} \tau_G, t_{zero}, \nu$<br>• Estimated only fixed effects of parameters: d_G<br>• Estimated only random effects of parameters $\log_{10} p, \delta, \log_{10} \beta, k,$	288.2<br>252.2

	Initial conditions:<br>$r_G = \frac{d_G I_G(0)}{V(0)}$ if $t < \tau_G$, else $r_G = R_G$	$log10(T(t_{zero}))$<br>• Error model: constant (IgG), constant (VL)<br>• No correlations among parameters	
MQ-v5	IgG binds to virus in density-dependent manner and there is continuous stimulation of B cells, which gets saturated at high levels of IgG,<br>$$\frac{dT}{dt} = -\beta VT$$<br>$$\frac{dI}{dt} = \beta VT - \delta I I^k$$<br>$$\frac{dV}{dt} = pI - cV - \nu I_G V$$<br>$$\frac{dI_G}{dt} = \frac{r_G I_G V}{k_G + I_G} - d_G I_G$$<br>Initial conditions:<br>$r_G = \frac{d_G(k_G + I_G(0))}{V(0)}$ if $t < \tau_G$, else $r_G = R_G$	• Estimated fixed and random effects of parameters:<br>$\log_{10} I_{G0}, r_G, \log_{10}\tau_G, t_{zero}, \nu$<br>• Estimated only fixed effects of parameters: k_G, d_G<br>• Estimated only random effects of parameters<br>$\log_{10} p, \delta, \log_{10}\beta, k,$<br>$log10(T(t_{zero}))$<br>• Error model: constant (IgG), constant (VL)<br>• No correlations among parameters	329.3<br>291.3
MQ-v6	IgG binds to virus in density-dependent manner and there is continuous stimulation of B cells, which gets saturated at high levels of viral loads ,<br>$$\frac{dT}{dt} = -\beta VT$$<br>$$\frac{dI}{dt} = \beta VT - \delta I I^k$$<br>$$\frac{dV}{dt} = pI - cV - \nu I_G V$$<br>$$\frac{dI_G}{dT} = \frac{r_G I_G V}{k_G + V} - d_G I_G$$<br>Initial conditions:<br>$r_G = \frac{d_G(k_G + V(0))}{V(0)}$ if $t < \tau_G$, else $r_G = R_G$	• Estimated fixed and random effects of parameters:<br>$\log_{10} I_{G0}, r_G, \log_{10}\tau_G, t_{zero}, \nu$<br>• Estimated only fixed effects of parameters: k_G, d_G<br>• Estimated only random effects of parameters<br>$\log_{10} p, \delta, \log_{10}\beta, k,$<br>$log10(T(t_{zero}))$<br>• Error model: constant (IgG), constant (VL)<br>• No correlations among parameters	280.0<br>242.0
MQ-v7	IgG binds to virus in density-dependent manner, IgG response to infection is τ days delayed and there is continuous stimulation of B cells,<br>$$\frac{dT}{dt} = -\beta VT$$<br>$$\frac{dI}{dt} = \beta VT - \delta I I^k$$<br>$$\frac{dV}{dt} = pI - cV - \nu I_G V$$<br>$$\frac{dI_G}{dt} = r_G V(t-\tau) - d_G I_G$$	• Estimated fixed and random effects of parameters:<br>$\log_{10} I_{G0}, r_G, \log_{10}\tau, t_{zero}, \nu$<br>• Estimated only fixed effects of parameters: k_G, d_G<br>• Estimated only random effects of parameters<br>$\log_{10} p, \delta, \log_{10}\beta, k,$<br>$log10(T(t_{zero}))$	314.9<br>258.9

		• Error model: constant (IgG), constant (VL)<br>• No correlations among parameters	
MQ-v8	IgG binds to virus in density-dependent manner, IgG response to infection is τ days delayed and there is continuous stimulation of B cells, which gets saturated at high levels of IgG,<br>$$\frac{dT}{dt} = -\beta VT$$<br>$$\frac{dI}{dt} = \beta VT - \delta I I^k$$<br>$$\frac{dV}{dt} = pI - cV - \nu I_G V$$<br>$$\frac{dI_G}{dt} = \frac{r_G I_G V(t-\tau)}{k_G + I_G} - d_G I_G$$	• Estimated fixed and random effects of parameters:<br>$\log_{10} I_{G0}, r_G, \log_{10} \tau, t_{zero}, \nu$<br>• Estimated only fixed effects of parameters: d_G<br>• Estimated only random effects of parameters<br>$\log_{10} p, \delta, \log_{10} \beta, k, log10(T(t_{zero}))$<br>• Error model: constant (IgG), constant (VL)<br>• No correlations among parameters	277.3<br>239.3
MQ-v9	IgG binds to virus in density-dependent manner, IgG response to infection is τ days delayed and there is continuous stimulation of B cells, which gets saturated at high levels of viral loads,<br>$$\frac{dT}{dt} = -\beta VT$$<br>$$\frac{dI}{dt} = \beta VT - \delta I I^k$$<br>$$\frac{dV}{dt} = pI - cV - \nu I_G V$$<br>$$\frac{dI_G}{dt} = \frac{r_G I_G V(t-\tau)}{k_G + V} - d_G I_G$$	• Estimated fixed and random effects of parameters:<br>$\log_{10} I_{G0}, r_G, \log_{10} \tau_G, t_{zero}, \nu$<br>• Estimated only fixed effects of parameters: d_G<br>• Estimated only random effects of parameters<br>$\log_{10} p, \delta, \log_{10} \beta, k, log10(T(t_{zero}))$<br>• Error model: constant (IgG), constant (VL)<br>No correlations among parameters	398.1<br>360.1
MR-v1	IgG affects viral entry and there is one-time stimulation of B cells<br>$$\frac{dT}{dt} = -\beta(1 - \frac{I_G}{k_E + I_G})VT$$<br>$$\frac{dI}{dt} = \beta(1 - \frac{I_G}{k_E + I_G})VT - \delta I I^k$$<br>$$\frac{dV}{dt} = pI - cV$$<br>$$\frac{dI_G}{dt} = r_G - d_G I_G$$<br>Initial conditions:<br>$r_G = d_G I_G(0)$ if $t < \tau_G$, else $r_G = R_G$	• Estimated fixed and random effects of parameters:<br>$\log_{10} I_{G0}, r_G, \log_{10} \tau_G, t_{zero}, k_E$<br>• Estimated only fixed effects of parameters: d_G<br>• Estimated only random effects of parameters<br>$\log_{10} p, \delta, \log_{10} \beta, k, log10(T(t_{zero}))$<br>• Error model: constant (IgG), constant (VL)<br>No correlations among parameters	233.0<br>197.0
MR-v2	IgG affects viral entry and there is one-time stimulation of B cells, which gets saturated by high levels of IgG,	• Estimated fixed and random effects of parameters:	214.1<br>176.1

	$\frac{dT}{dt} = -\beta(1-\frac{I_G}{k_E+I_G})VT$<br>$\frac{dI}{dt} = \beta(1-\frac{I_G}{k_E+I_G})VT - \delta I I^k$<br>$\frac{dV}{dt} = pI - cV$<br>$\frac{dI_G}{dt} = \frac{r_G I_G}{k_G+I_G} - d_G I_G$<br>Initial conditions:<br>$r_G = d_G(k_G + I_G(0))$ if $t < \tau_G$, else $r_G = R_G$	$\log_{10} I_{G0}, r_G, \log_{10}\tau_G,\ t_{zero}, k_E$<br>• Estimated only fixed effects of parameters: k_G, d_G<br>• Estimated only random effects of parameters $\log_{10} p, \delta, \log_{10}\beta, k, log10(T(t_{zero}))$<br>• Error model: constant (IgG), constant (VL)<br>No correlations among parameters	
MR-v3	IgG affects viral entry and there is one-time stimulation of B cells, which gets saturated by high levels of viral loads,<br>$\frac{dT}{dt} = -\beta(1-\frac{I_G}{k_E+I_G})VT$<br>$\frac{dI}{dt} = \beta(1-\frac{I_G}{k_E+I_G})VT - \delta I I^k$<br>$\frac{dV}{dt} = pI - cV$<br>$\frac{dI_G}{dt} = \frac{r_G I_G}{k_G+V} - d_G I_G$<br>Initial conditions:<br>$r_G = d_G(k_G + V(0))$ if $< \tau_G$, else $r_G = R_G$	• Estimated fixed and random effects of parameters:<br>$\log_{10} I_{G0}, r_G, \log_{10}\tau_G,\ t_{zero}, k_E$<br>• Estimated only fixed effects of parameters: k_G, d_G<br>• Estimated only random effects of parameters $\log_{10} p, \delta, \log_{10}\beta, k, log10(T(t_{zero}))$<br>• Error model: constant (IgG), constant (VL)<br>No correlations among parameters	419.6<br>381.6
MR-v4	IgG affects viral entry and there is continuous stimulation of B cells<br>$\frac{dT}{dt} = -\beta(1-\frac{I_G}{k_E+I_G})VT$<br>$\frac{dI}{dt} = \beta(1-\frac{I_G}{k_E+I_G})VT - \delta I I^k$<br>$\frac{dV}{dt} = pI - cV$<br>$\frac{dI_G}{dt} = r_G V - d_G I_G$<br>Initial conditions:<br>$r_G = \frac{d_G I_G(0)}{V(0)} if\ t < \tau_G, else\ r_G = R_G$	• Estimated fixed and random effects of parameters:<br>$\log_{10} I_{G0}, r_G, \log_{10}\tau_G,\ t_{zero}, k_E$<br>• Estimated only fixed effects of parameters: d_G<br>• Estimated only random effects of parameters $\log_{10} p, \delta, \log_{10}\beta, k, log10(T(t_{zero}))$<br>• Error model: constant (IgG), constant (VL)<br>• No correlations among parameters	312.7<br>276.7
MR-v5	IgG affects viral entry and there is continuous stimulation of B cells, which gets saturated by high levels of IgG,<br>$\frac{dT}{dt} = -\beta(1-\frac{I_G}{k_E+I_G})VT$<br>$\frac{dI}{dt} = \beta(1-\frac{I_G}{k_E+I_G})VT - \delta I I^k$	• Estimated fixed and random effects of parameters:<br>$\log_{10} I_{G0}, r_G, \log_{10}\tau_G,\ t_{zero}, k_E$<br>• Estimated only fixed effects of parameters: k_G, d_G	321.5<br>283.5

	$$\frac{dV}{dt} = pI - cV$$<br>$$\frac{dI_G}{dt} = \frac{r_G I_G V}{k_G + I_G} - d_G I_G$$<br>Initial conditions:<br>$r_G = \frac{d_G(k_G + I_G(0))}{V(0)}$ if $t < \tau_G$, else $r_G = R_G$	• Estimated only random effects of parameters $\log_{10} p, \delta, \log_{10} \beta, k, log10(T(t_{zero}))$<br>• Error model: constant (IgG), constant (VL)<br>• No correlations among parameters	
MR-v6	IgG affects viral entry and there is continuous stimulation of B cells, which gets saturated by high levels of viral loads,<br>$$\frac{dT}{dt} = -\beta(1 - \frac{I_G}{k_E + I_G})VT$$<br>$$\frac{dI}{dt} = \beta(1 - \frac{I_G}{k_E + I_G})VT - \delta I I^k$$<br>$$\frac{dV}{dt} = pI - cV$$<br>$$\frac{dI_G}{dT} = \frac{r_G I_G V}{k_G + V} - d_G I_G$$<br>Initial conditions:<br>$r_G = \frac{d_G(k_G + V(0))}{V(0)}$ if $t < \tau_G$, else $r_G = R_G$	• Estimated fixed and random effects of parameters:<br>$\log_{10} I_{G0}, r_G, \log_{10} \tau_G, t_{zero}, k_E$<br>• Estimated only fixed effects of parameters: k_G, d_G<br>• Estimated only random effects of parameters $\log_{10} p, \delta, \log_{10} \beta, k, log10(T(t_{zero}))$<br>• Error model: constant (IgG), constant (VL)<br>• No correlations among parameters	273.8<br>235.8
MR-v7	IgG affects VL entry, IgG response to infection is τ days delayed and there is continuous stimulation of B cells,<br>$$\frac{dT}{dt} = -\beta(1 - \frac{I_G}{k_E + I_G})VT$$<br>$$\frac{dI}{dt} = \beta(1 - \frac{I_G}{k_E + I_G})VT - \delta I I^k$$<br>$$\frac{dV}{dt} = pI - cV$$<br>$$\frac{dI_G}{dt} = r_G V(t - \tau) - d_G I_G$$	• Estimated fixed and random effects of parameters:<br>$\log_{10} I_{G0}, r_G, \log_{10} \tau, t_{zero}, k_E$<br>• Estimated only fixed effects of parameters: k_G, d_G<br>• Estimated only random effects of parameters $\log_{10} p, \delta, \log_{10} \beta, k, log10(T(t_{zero}))$<br>• Error model: constant (IgG), constant (VL)<br>No correlations among parameters	294.4<br>258.4
MR-v8	IgG affects VL entry, IgG response to infection is τ days delayed and there is continuous stimulation of B cells, which gets saturated by high levels of IgG,<br>$$\frac{dT}{dt} = -\beta(1 - \frac{I_G}{k_E + I_G})VT$$<br>$$\frac{dI}{dt} = \beta(1 - \frac{I_G}{k_E + I_G})VT - \delta I I^k$$	• Estimated fixed and random effects of parameters:<br>$\log_{10} I_{G0}, r_G, \log_{10} \tau, t_{zero}, k_E$<br>• Estimated only fixed effects of parameters: d_G<br>• Estimated only random effects of parameters $\log_{10} p, \delta, \log_{10} \beta, k,$	287.3<br>249.3

	$$\frac{dV}{dt} = pI - cV$$ $$\frac{dI_G}{dt} = \frac{r_G I_G V(t-\tau)}{k_G + I_G} - d_G I_G$$	$log10(T(t_{zero}))$<br>• Error model: constant (IgG), constant (VL)<br>No correlations among parameters	
MR-v9	IgG affects VL entry, IgG response to infection is τ days delayed and there is continuous stimulation of B cells, which gets saturated by high levels of viral loads, $$\frac{dT}{dt} = -\beta(1 - \frac{I_G}{k_E + I_G})VT$$ $$\frac{dI}{dt} = \beta(1 - \frac{I_G}{k_E + I_G})VT - \delta I I^k$$ $$\frac{dV}{dt} = pI - cV$$ $$\frac{dI_G}{dt} = \frac{r_G I_G V(t-\tau)}{k_G + V} - d_G I_G$$	• Estimated fixed and random effects of parameters:<br>$\log_{10} I_{G0}, r_G, \log_{10} \tau_G, t_{zero}, k_E$<br>• Estimated only fixed effects of parameters: d_G<br>• Estimated only random effects of parameters<br>$\log_{10} p, \delta, \log_{10} \beta, k, log10(T(t_{zero}))$<br>• Error model: constant (IgG), constant (VL)<br>No correlations among parameters	330.0<br>292.0
MS-v1	IgG affect viral entry, binds to virus in density-dependent manner and there is one-time stimulation of B cells, $$\frac{dT}{dt} = -\beta(1 - \frac{I_G}{k_E + I_G})VT$$ $$\frac{dI}{dt} = \beta(1 - \frac{I_G}{k_E + I_G})VT - \delta I I^k$$ $$\frac{dV}{dt} = pI - cV - \nu I_G V$$ $$\frac{dI_G}{dt} = r_G - d_G I_G$$ Initial Conditions:<br>$r_G = d_G I_G(0)$ if $t < \tau_G$, else $r_G = R_G$	• Estimated fixed and random effects of parameters:<br>$\log_{10} I_{G0}, r_G, \log_{10} \tau_G, t_{zero}, k_E, \nu$<br>• Estimated only fixed effects of parameters: d_G<br>• Estimated only random effects of parameters<br>$\log_{10} p, \delta, \log_{10} \beta, k, log10(T(t_{zero}))$<br>• Error model: constant (IgG), constant (VL)<br>No correlations among parameters	238.0<br>198.0
MS-v2	IgG affect viral entry, binds to virus in density-dependent manner and there is one-time stimulation of B cells, which gets saturated by high levels of IgG, $$\frac{dT}{dt} = -\beta(1 - \frac{I_G}{k_E + I_G})VT$$ $$\frac{dI}{dt} = \beta(1 - \frac{I_G}{k_E + I_G})VT - \delta I I^k$$ $$\frac{dV}{dt} = pI - cV - \nu I_G V$$ $$\frac{dI_G}{dt} = \frac{r_G I_G}{k_G + I_G} - d_G I_G$$ Initial Conditions:<br>$r_G = d_G(k_G + I_G(0))$ if $t < \tau_G$, else $r_G = R_G$	• Estimated fixed and random effects of parameters:<br>$\log_{10} I_{G0}, r_G, \log_{10} \tau_G, t_{zero}, k_E, \nu$<br>• Estimated only fixed effects of parameters: k_G, d_G<br>• Estimated only random effects of parameters<br>$\log_{10} p, \delta, \log_{10} \beta, k, log10(T(t_{zero}))$<br>• Error model: combined1 (IgG), constant (VL)<br>No correlations among	212.4<br>168.4

		parameters	
MS-v3	IgG affect viral entry, binds to virus in density-dependent manner and there is one-time stimulation of B cells, which gets saturated by high levels of viral loads, $$\frac{dT}{dt} = -\beta(1-\frac{I_G}{k_E+I_G})VT$$ $$\frac{dI}{dt} = \beta(1-\frac{I_G}{k_E+I_G})VT - \delta I I^k$$ $$\frac{dV}{dt} = pI - cV - \nu I_G V$$ $$\frac{dI_G}{dt} = \frac{r_G I_G}{k_G+V} - d_G I_G$$ Initial Conditions: $r_G = d_G(k_G + V(0))$ if $t < \tau_G$, else $r_G = R_G$	• Estimated fixed and random effects of parameters: $\log_{10} I_{G0}, r_G, \log_{10}\tau_G, t_{zero}, k_E, \nu$<br>• Estimated only fixed effects of parameters: k_G, d_G<br>• Estimated only random effects of parameters $\log_{10} p, \delta, \log_{10}\beta, k, log10(T(t_{zero}))$<br>• Error model: constant (IgG), constant (VL) No correlations among parameters	409.6<br>367.6
MS-v4	IgG affect viral entry, binds to virus in density-dependent manner and there is continuous stimulation of B cells, $$\frac{dT}{dt} = -\beta(1-\frac{I_G}{K_E+I_G})VT$$ $$\frac{dI}{dt} = \beta(1-\frac{I_G}{k_E+I_G})VT - \delta I I^k$$ $$\frac{dV}{dt} = pI - cV - \nu I_G V$$ $$\frac{dI_G}{dt} = r_G V - d_G I_G$$ Initial Conditions: $r_G = \frac{d_G I_G(0)}{V(0)}$ if $t < \tau_G$, else $r_G = R_G$	• Estimated fixed and random effects of parameters: $\log_{10} I_{G0}, r_G, \log_{10}\tau_G, t_{zero}, k_E, \nu$<br>• Estimated only fixed effects of parameters: d_G<br>• Estimated only random effects of parameters $\log_{10} p, \delta, \log_{10}\beta, k, log10(T(t_{zero}))$<br>• Error model: constant (IgG), constant (VL)<br>• No correlations among parameters<br>•	320.5<br>280.5
MS-v5	IgG affect viral entry, binds to virus in density-dependent manner and there is continuous stimulation of B cells, which gets saturated by high levels of IgG, $$\frac{dT}{dt} = -\beta(1-\frac{I_G}{k_E+I_G})VT$$ $$\frac{dI}{dt} = \beta(1-\frac{I_G}{k_E+I_G})VT - \delta I I^k$$ $$\frac{dV}{dt} = pI - cV - \nu I_G V$$ $$\frac{dI_G}{dt} = \frac{r_G I_G V}{k_G+I_G} - d_G I_G$$ Initial Conditions: $r_G = \frac{d_G(k_G+I_G)}{V(0)}$ if $t < \tau_G$, else $r_G = R_G$	• Estimated fixed and random effects of parameters: $\log_{10} I_{G0}, r_G, \log_{10}\tau_G, t_{zero}, k_E, \nu$<br>• Estimated only fixed effects of parameters: k_G, d_G<br>• Estimated only random effects of parameters $\log_{10} p, \delta, \log_{10}\beta, k, log10(T(t_{zero}))$<br>• Error model: constant (IgG), constant (VL)<br>• No correlations among parameters	324.7<br>282.7

MS-v6	IgG affect viral entry, binds to virus in density-dependent manner and there is continuous stimulation of B cells, which gets saturated by high levels of viral loads,<br>$\frac{dT}{dt} = -\beta(1-\frac{I_G}{k_E+I_G})VT$<br>$\frac{dI}{dt} = \beta(1-\frac{I_G}{k_E+I_G})VT - \delta I I^k$<br>$\frac{dV}{dt} = pI - cV - \nu I_G V$<br>$\frac{dI_G}{dT} = \frac{r_G I_G V}{k_G+V} - d_G I_G$<br>Initial Conditions:<br>$r_G = \frac{d_G(k_G+V(0))}{V(0)}$ if $t < \tau_G$, else $r_G = R_G$	• Estimated fixed and random effects of parameters: $\log_{10} I_{G0}, r_G, \log_{10}\tau_G, t_{zero}, k_E, \nu$<br>• Estimated only fixed effects of parameters: k_G, d_G<br>• Estimated only random effects of parameters $\log_{10} p, \delta, \log_{10}\beta, k, log10(T(t_{zero}))$<br>• Error model: constant (IgG), constant (VL)<br>• No correlations among parameters	267.1<br>225.1
MS-v7	IgG affect viral entry, binds to virus in density-dependent manner, IgG response to infection is τ days delayed and there is continuous stimulation of B cells,<br>$\frac{dT}{dt} = -\beta(1-\frac{I_G}{k_E+I_G})VT$<br>$\frac{dI}{dt} = \beta(1-\frac{I_G}{k_E+I_G})VT - \delta I I^k$<br>$\frac{dV}{dt} = pI - cV - \nu I_G V$<br>$\frac{dI_G}{dt} = r_G V(t-\tau) - d_G I_G$	• Estimated fixed and random effects of parameters: $\log_{10} I_{G0}, r_G, \log_{10}\tau_G, t_{zero}, k_E, \nu$<br>• Estimated only fixed effects of parameters: k_G, d_G<br>• Estimated only random effects of parameters $\log_{10} p, \delta, \log_{10}\beta, k, log10(T(t_{zero}))$<br>• Error model: constant (IgG), constant (VL)<br>No correlations among parameters	306.3<br>266.3
MS-v8	IgG affect viral entry, binds to virus in density-dependent manner, IgG response to infection is τ days delayed and there is continuous stimulation of B cells, which gets saturated by high levels of IgG,<br>$\frac{dT}{dt} = -\beta(1-\frac{I_G}{k_E+I_G})VT$<br>$\frac{dI}{dt} = \beta(1-\frac{I_G}{k_E+I_G})VT - \delta I I^k$<br>$\frac{dV}{dt} = pI - cV - \nu I_G V$<br>$\frac{dI_G}{dt} = \frac{r_G I_G V(t-\tau)}{k_G+I_G} - d_G I_G$	• Estimated fixed and random effects of parameters: $\log_{10} I_{G0}, r_G, \log_{10}\tau_G, t_{zero}, k_E, \nu$<br>• Estimated only fixed effects of parameters: d_G<br>• Estimated only random effects of parameters $\log_{10} p, \delta, \log_{10}\beta, k, log10(T(t_{zero}))$<br>• Error model: constant (IgG), constant (VL)<br>No correlations among parameters	280.6<br>238.6

MS-v9	IgG affect viral entry, binds to virus in density-dependent manner, IgG response to infection is τ days delayed and there is continuous stimulation of B cells, which gets saturated by high levels of viral loads,<br>$\frac{dT}{dt} = -\beta(1 - \frac{I_G}{k_E + I_G})VT$<br>$\frac{dI}{dt} = \beta(1 - \frac{I_G}{k_E + I_G})VT - \delta I I^k$<br>$\frac{dV}{dt} = pI - cV - \nu I_G V$<br>$\frac{dI_G}{dt} = \frac{r_G I_G V(t-\tau)}{k_G + V} - d_G I_G$	• Estimated fixed and random effects of parameters:<br>$\log_{10} I_{G0}, r_G, \log_{10} \tau_G, t_{zero}, k_E, \nu$<br>• Estimated only fixed effects of parameters: d_G<br>• Estimated only random effects of parameters<br>$\log_{10} p, \delta, \log_{10} \beta, k, log10(T(t_{zero}))$<br>• Error model: constant (IgG), constant (VL)<br>No correlations among parameters	332.4<br>290.4
MT-v1	IgG induces death of infected cells and there is one-time stimulation of B cells,<br>$\frac{dT}{dt} = -\beta VT$<br>$\frac{dI}{dt} = \beta VT - \delta I I^k - \phi I I_G$<br>$\frac{dV}{dt} = pI - cV$<br>$\frac{dI_G}{dt} = r_G - d_G I_G$<br>Initial conditions:<br>$r_G = d_G I_G(0)$ if $t < \tau_G$, else $r_G = R_G$	• Estimated fixed and random effects of parameters:<br>$\log_{10} I_{G0}, r_G, \log_{10} \tau_G, t_{zero}, \phi$<br>• Estimated only fixed effects of parameters: d_G<br>• Estimated only random effects of parameters<br>$\log_{10} p, \delta, \log_{10} \beta, k, log10(T(t_{zero}))$<br>• Error model: constant (IgG), constant (VL)<br>• No correlations among parameters	234.3<br>198.3
MT-v2	IgG induces death of infected cells and there is one-time stimulation of B cells, which gets saturated at the high concentration of IgG,<br>$\frac{dT}{dt} = -\beta VT$<br>$\frac{dI}{dt} = \beta VT - \delta I I^k - \phi I I_G$<br>$\frac{dV}{dt} = pI - cV$<br>$\frac{dI_G}{dt} = \frac{r_G I_G}{k_G + I_G} - d_G I_G$<br>Initial conditions:<br>$r_G = d_G(k_G + I_G(0))$ if $t < \tau_G$, else $r_G = R_G$	• Estimated fixed and random effects of parameters:<br>$\log_{10} I_{G0}, r_G, \log_{10} \tau_G, t_{zero}, \phi$<br>• Estimated only fixed effects of parameters: k_G, d_G<br>• Estimated only random effects of parameters<br>$\log_{10} p, \delta, \log_{10} \beta, k, log10(T(t_{zero}))$<br>• Error model: constant (IgG), constant (VL)<br>• Corr(R_G, δ)=0.999	209.1<br>169.1

Table S4: Estimated individual parameters under the model recapitulating longitudinal viral loads and IgG dynamics (MP-v2 in Table S3). Parameters d_G and k_G are estimated at 0.46/day and 9.3, respectively. Here, NA represents not available.

ID	$\log_{10} I_{G0}$ (SCO)	$\log_{10} p$ (/day)	δ (day-	$\log_{10} \tau$ (days)	R_G SCO/da	t_{zero} (days)	$\log_{10} \beta$	$\log_{10} T(t_{zero})$	k

			1·cells-k)		y				
1	0.02	2.61	0.67	0.67	22.3	-2.9	-7.18	7.05	0.15
2	0.02	2.58	0.43	0.89	55.7	-2.6	-7.25	6.99	0.07
3	0.02	2.59	0.49	0.78	59.3	-2.7	-7.23	7.00	0.07
4	0.02	2.59	0.52	0.77	8.7	-2.7	-7.23	7.00	0.10
5	0.02	2.60	5.56	-0.54	76.1	-2.6	-7.21	7.02	0.06
6	0.02	2.59	0.28	1.11	70.5	-2.7	-7.23	7.00	0.08
Median	0.02	2.59	0.50	0.77	57.5	-2.7	-7.23	7.0	0.08
Mean	0.02	2.59	1.32	0.61	48.7	-2.7	-7.22	7.0	0.09
95%CI	[-0.002, 0.04]	NA	NA	[0.20, 0.98]	[20.0, 68.8]	NA	NA	NA	NA

Table S5: Estimated individual parameters under the model recapitulating longitudinal viral loads and IgG dynamics from two additional patients from a different study [6] using model MP-v2 in Table S3. We fixed $d_G = 0.46$/day while parameter k_G was fixed at 9.3.

ID	$\log_{10} I_{G0}$ (SCO)	$\log_{10} p$ (/day)	δ (day^{-1}·cells^{-k})	$\log_{10} \tau$ (days)	R_G SCO/day	t_{zero} (days)	$\log_{10} \beta$	$\log_{10} T(t_{zero})$	k
7	0.08	2.57	0.20	1.30	15.8	-2.45	-7.25	6.98	0.09
8	0.07	2.50	0.91	0.82	5.9	-2.53	-7.26	6.85	0.09

References

1. Nutt, S.L., et al., *The generation of antibody-secreting plasma cells.* Nat Rev Immunol, 2015. **15**(3): p. 160-71.
2. Appelgren, D., et al., *Marginal-Zone B-Cells Are Main Producers of IgM in Humans, and Are Reduced in Patients With Autoimmune Vasculitis.* Front Immunol, 2018. **9**(2242): p. 2242.
3. Zou, Y.-R., C. Grimaldi, and B. Diamond, *B Cells*, in *Kelley and Firestein's Textbook of Rheumatology*, G.S. Firestein, et al., Editors. 2017, Elsevier. p. 207-230.e3.
4. Hoffman, W., F.G. Lakkis, and G. Chalasani, *B Cells, Antibodies, and More.* Clin J Am Soc Nephrol, 2016. **11**(1): p. 137-54.
5. Goyal, A., E.F. Cardozo-Ojeda, and J.T. Schiffer, *Potency and timing of antiviral therapy as determinants of duration of SARS-CoV-2 shedding and intensity of inflammatory response.* Sci Adv, 2020. **6**(47): p. eabc7112.
6. Cervia, C., et al., *Systemic and mucosal antibody responses specific to SARS-CoV-2 during mild versus severe COVID-19.* J Allergy Clin Immunol, 2020.
